# Increasing Response Vigour Under Time Pressure as a Transdiagnostic Marker of Eating Disorders

**DOI:** 10.5334/cpsy.130

**Published:** 2025-08-14

**Authors:** Sam Hall-McMaster, Ondrej Zika

**Affiliations:** 1 Max Planck Institute for Human Development, Germany; 2 Harvard University, United States; 3 Faculty of Psychology and Sports Science, Department of Psychology, Biological and Cognitive Neurosciences, Bielefeld University, Germany; 4 Centre for Psychiatry Research, Department of Clinical Neuroscience, Karolinska Institutet, & Stockholm Health Care Services, Region Stockholm, Sweden; 5 Department of Clinical Psychology and Psychotherapy, Babeș-Bolyai University, Cluj-Napoca, Romania

**Keywords:** decision-making, foraging, eating disorders

## Abstract

Eating disorders (EDs) are characterised by intense concerns about food and weight. These concerns are linked to changes in decision-making, such as persisting with actions that are no longer rewarding. For example, individuals might engage in long exercise sessions or time-consuming body checking practices, despite limited benefits. This study tested whether people with subclinical ED symptoms show increased persistence due to altered decision-making processes. Specifically, we postulated a shift in internal thresholds for making different decisions in EDs, which change the balance between exploitation and exploration. A subclinical group with heightened concerns about eating (sED; N = 44) and a healthy control group (HC; N = 56) completed a foraging task, in which an option on screen was exploited for reward. With each decision to exploit, reward feedback decreased and participants had to decide when to move on to a new option. Each block was time limited to 7.5 minutes. Behavioural persistence was measured as the number of seconds spent exploiting each option. Decision thresholds were measured when deciding to move on, as the counterfactual reward that would have been received for an exploit action. We predicted that the sED group would show increased persistence and decreased decision thresholds (i.e. lower counterfactual reward when deciding to move on) in comparison to the HC group. We found no evidence for these predictions. Instead, exploratory analyses showed that the sED group exhibited progressively faster response times (RTs) when approaching the time limit for each block. This increase in motor vigour was correlated with the severity of eating disorder symptoms from a range of traditional diagnostic categories. Our results point to changing motor vigour as a potential transdiagnostic marker of ED tendencies.

## Introduction

Eating disorders (EDs) encompass a range of heterogeneous conditions, including anorexia nervosa (AN), bulimia nervosa and binge eating disorder (American Psychiatric Association, 2013). The behavioural symptoms often associated with these conditions, such as preoccupation with food and weight, are also common in the general population (Abdulkadir, et al., 2022; Romano et al., 2022). It is no surprise that these concerns can influence food-related choices. For example, people with acute AN have been shown to choose high fat foods significantly less than people who are otherwise healthy (Foerde et al., 2015), a pattern of decision-making that persists even after weight has been restored (Foerde et al., 2021). An intriguing observation from ED studies, particularly those focused on restrictive EDs, is that decisions seemingly unrelated to food can be affected too (Bernardoni et al., 2018; Bernardoni et al., 2021; Chan et al., 2014; Decker et al., 2015; Foerde et al., 2021; Guillaume et al., 2015; Jenkinson et al., 2023; Onysk & Seriès, 2022; Pike et al., 2023; Steinglass et al., 2006; Steinglass et al., 2012; Steinglass et al., 2017; Radzikowska et al., 2025; Schuman et al., 2025; Verharen et al., 2019). For example, individuals with AN perform worse in probabilistic choice tasks that use neutral stimuli than matched controls (Chan et al., 2014; Verharen et al., 2019), and individuals with subclinical AN symptoms are more willing to pick delayed monetary rewards over immediate ones (Schuman et al., 2025). Observations along these lines suggest that general-purpose decision mechanisms may be altered in restrictive EDs, at least for certain classes of decisions and/or at certain stages of illness.

Most studies to date on cognition in restrictive EDs have focused on a specific class of decision problem, in which individuals make independent choices between simultaneously presented options (Bernardoni et al., 2018; Bernardoni et al., 2021; Chan et al., 2014; Decker et al., 2015; Foerde et al., 2015; Pike et al., 2023; Rouhani et al., 2025; Steinglass et al., 2006; Steinglass et al., 2012; Steinglass et al., 2017; Schuman et al., 2025; Verharen et al., 2019). Examples include the food choice task, where people choose between pairs of food items (Foerde et al., 2015; Foerde et al., 2018) and reinforcement learning tasks where people choose between pairs of abstract stimuli (Bernardoni et al., 2018; Bernardoni et al., 2021; Pike et al., 2023). In cases like these, a key property is choice independence: one’s actions in the current trial do not influence what is shown on the next trial. While there is substantial evidence that responses to independent choice problems are altered in restrictive EDs (Bernardoni et al., 2018; Bernardoni et al., 2021; Chan et al., 2014; Decker et al., 2015; Foerde et al., 2015; Pike et al., 2023; Rouhani et al., 2025; Steinglass et al., 2006; Steinglass et al., 2012; Steinglass et al., 2017; Schuman et al., 2025; Verharen et al., 2019), there is a growing interest in choice problems that have sequential dependence, where the actions taken influence the subsequent decisions one is faced with (Foerde et al., 2021; Jenkinson et al., 2023; Onysk & Seriès, 2022).

To illustrate why this is relevant to the study of restrictive EDs, imagine making a decision about how long to continue exercising before stopping, or whether to continue fasting when initial feelings of hunger arise. Decisions in these cases exhibit sequential dependence. If you decide to keep exercising, you will be faced with a similar choice later on. In such cases, one intuition is that someone with restrictive eating tendencies may be motivated to persist for longer than the general population, exercising for longer before stopping or fasting for longer before eating. Indeed, current evidence suggests that exercise duration increases with restrictive eating symptoms (Rizk et al., 2015) and individuals with AN continue using decision rules once they become irrelevant, longer than healthy controls do (Filoteo et al., 2014; Steinglass et al., 2006; Wu et al., 2014). One hypothesis that emerges from these findings is that people with restrictive EDs struggle to disengage from actions or activities, even when it would be beneficial. This could potentially contribute to a range of behaviours, including longer exercise sessions in individuals with AN (Rizk et al., 2015), more time washing and getting dressed in AN compared to other EDs (St-Pierre et al., 2023), time-consuming body checking practices that can occur in AN or atypical AN (Harrop et al., 2023), and meal skipping in subclinical individuals at risk of developing an ED (Kabakuş Aykut & Bilici, 2022).

Computational studies of restrictive EDs suggest that difficulties adapting behaviour when needed could arise from a combination of reduced exploratory behaviour and increased perseveration (Filoteo et al., 2014). Reduced risk aversion could also contribute in certain cases, increasing persistence when actions lead to illness-consistent outcomes (Jenkinson et al., 2023). In addition to these changes, decision-making in AN and subclinical EDs becomes less model-based (Foerde et al., 2021; Onysk & Seriès, 2022), a form of behavioural control that normally supports flexible adjustment to changing circumstances (Voon et al., 2017). Based on current evidence, there are several candidate mechanisms that could contribute to maladaptive persistence in restrictive EDs.

One theoretical possibility that has not been considered is an altered threshold for adapting behaviour. This idea is formalised in Optimal Foraging Theory (OFT; Stephens & Krebs, 1986). OFT was originally developed to understand how animals search for food in natural environments (Calhoun & Hayden, 2015; Hayden & Walton, 2014; Mobbs et al., 2018), but has since been adopted to understand how people make decisions (Bustamante et al., 2023; Constantino and Daw, 2015; Hall-McMaster et al., 2021; Harhen & Bornstein, 2023; Kolling et al., 2012; Le Heron et al., 2020; Wilke et al., 2009; Razza et al., 2025; Wittmann et al., 2016). A central dilemma considered in OFT is patch-leaving, in which one must decide when to leave an option with diminishing returns (Stephens & Krebs, 1986; Stephens, 2008). Patch-leaving has conceptual links to the explore-exploit dilemma (Addicott et al., 2017; Hagan et al., 2024), where it has been proposed that a bias to over-exploit could lead to persistent restriction in EDs (Hagan et al., 2024). Under certain conditions, the normative solution to the patch-leaving problem is to leave the current option when its reward falls below a decision threshold, set to the average environmental reward rate (Charnov, 1976; Hayden et al., 2011). This implies that failures to adapt could be due to an abnormal decision threshold that is only met after excessive exploitation. In the case of deciding how long to exercise, for example, the diminishing returns of continued exercise would have to drop to an especially low level before a person decides to stop.

Within psychiatry, OFT has been previously applied in studies of addiction (Addicott et al., 2015; Raio et al., 2022). Using patch-leaving tasks, these studies have measured the time spent exploiting each option (persistence), reward levels when deciding to leave each option (decision thresholds), and how much these quantities deviate from an optimal agent or an empirical control group. This approach has been fruitful, revealing altered decision thresholds in opioid addiction and gambling addiction respectively (Addicott et al., 2015; Raio et al., 2022). So far, OFT has not been used to examine failures to adapt in individuals with restrictive ED symptoms. Here we propose that such failures could arise from altered decision thresholds, a prediction that can be tested using patch-leaving. Altered thresholds themselves could stem from altered reward learning processes reported in restrictive EDs (e.g. Foerde & Steinglass, 2017; Wierenga et al., 2021) that lower internal estimates of the average environmental reward rate. This possibility can be assessed with reinforcement learning models, which are often used to understand latent processes that contribute to foraging behaviour (Constantino & Daw, 2015; Frankenhuis et al., 2019; Hall-McMaster & Luyckx, 2019; Kolling & Akam, 2017; Wittmann et al., 2016).

Based on research suggesting maladaptive persistence in restrictive EDs (Filoteo et al., 2014; Steinglass et al., 2006; Wu et al., 2014; Rizk et al., 2015), the present experiment aimed to test whether individuals with restrictive ED tendencies show increased persistence due to altered decision thresholds for adapting their behaviour. Based on Optimal Foraging Theory, we predicted this would be seen in a computerised foraging task as: 1) more time spent exploiting each option before deciding to disengage from it (increased persistence) in comparison to HCs and 2) continuing to exploit options to lower levels of reward before disengaging (a decreased decision threshold) in comparison to HCs. The experiment was conducted using a subclinical online sample, with dieting and oral control subscales of the Eating Attitudes Test (EAT-26; Gardner et al., 1982; Berland et al., 1986) being used as indicators of restrictive ED tendencies.

## Methods

### Participants

The final sample had a 44 participant subclinical ED (sED) group (mean age = 25, mean years of education = 16, mean BMI = 22.04, 40 female, 4 male), and a 56 participant control group (mean age = 27, mean years of education = 17, mean BMI = 22.74, 45 female, 11 male). Participants were separated into groups based on scores from the EAT-26 (Garner et al., 1982), where higher scores indicate greater concerns about eating. Those in the sED group had scores of 20 or higher (mean = 36.05), whereas those in the control group had scores below 10 (mean = 3.13). The sED cutoff of 20 was based on the EAT-26 scoring rubric, which proposes that a score ≥ 20 indicates a “high level of concern about dieting, body weight, or problematic eating behaviours”. Individuals with scores ≥ 20 score are recommended to “seek an evaluation by a qualified health professional” to determine if their score “reflects a problem that warrants clinical attention” (Garner et al. 1982). The cutoff for the control group was from Onysk & Seriès (2022). Eleven people in the sED group indicated they had a formal ED diagnosis, compared with zero individuals in the control group (six individuals across both groups chose not to report this information). Participants were fluent in English and did not report using medication for a neurological or psychiatric condition. Height, weight, age, gender and years of education were self-reported. Participants received £10.10 for completing the full experiment, as well as a bonus of up to £2 based on performance in the decision task. Baseline compensation was distributed across the experimental stages as: £0.6 for the prescreening, £4 for the bigger questionnaire battery, and £5.50 for the decision task. The study was approved by the ethics committee at the Max Planck Institute for Human Development (i2023-03) and participants gave informed consent before taking part.

### Materials

**Figure 1:**
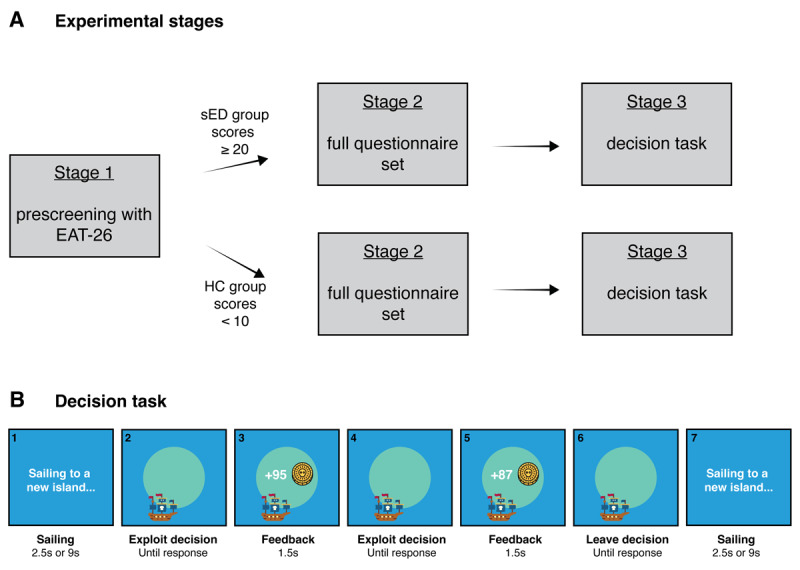
Experimental stages and task design. A: The experiment proceeded in three main stages. Prospective participants were first invited to complete the Eating Attitudes Test (Garner et al., 1982). Participants were sorted based on their scores. Those with scores of 20 or higher were assigned to the subclinical eating disorder (sED) group and those with scores below 10 were assigned to the healthy control (HC) group. From this stage on, the groups proceeded in parallel tracks. Participants from each group were invited to complete a full questionnaire set that assessed eating behaviours and attitudes, body image, anxiety and depression, as well as intolerance for uncertainty and obsessive-compulsive tendencies. In the final stage, participants were invited to complete a decision task. B: During the decision task, participants sailed to islands to dig for buried treasure. Sailing to an island involved a 2.5s or 9s delay. After arriving, participants could choose between an exploit action (digging) or leaving to sail to a new island. An exploit action resulted in reward feedback, after which participants faced a new decision about whether to exploit or leave. With each successive exploit action on the current island, the reward decreased (fast decay rate = 0.81; slow decay rate = 0.91). Across blocks, we manipulated the travel time between islands and how quickly the reward depleted.

Participants completed a series of questionnaires about eating attitudes and mental health. These included the EAT-26 (Garner et al., 1982), Eating Disorder Examination Questionnaire (EDE-Q; Fairburn & Beglin, 2008), Appearance Anxiety Inventory (AAI; Veale et al., 2014), Clinical Impairment Assessment (CIA; Bohn & Fairburn, 2008), State-Trait Anxiety Inventory (STAI; Spielberger, 1983), Beck Depression Inventory (BDI-II; Beck et al., 1996), Intolerance for Uncertainty Scale (IUS; full version; Buhr & Dugas, 2002), and the Obsessive-Compulsive Inventory (OCI-R; revised version; Foa et al., 2002). Questionnaires were presented in Limesurvey (https://www.limesurvey.org/). Participants also completed a cognitive task, built in Psychopy-3 (https://www.psychopy.org; Peirce et al., 2019) and hosted on Pavlovia (https://pavlovia.org/). The task included two cartoon stimuli that were created by icon developers Smalllikeart and Nikita Golubev (accessed from https://www.flaticon.com). Arrows in Figure 1 were created by Atif Arshad (accessed from https://www.flaticon.com). Participants were recruited for the experiment using Prolific (https://www.prolific.co/) and tested online. Analyses were conducted in Python3.

### Procedure

The final sample was reached using a three-step procedure. In the first step, prospective participants completed the EAT-26. Following Onysk and Seriès (2022), Prolific’s prescreening tools were used to distribute the EAT-26 to relevant participants. For the sED group, invitations were sent to people who reported: 1) going on a diet in the past; 2) restricting their food intake for the last week or longer, to lose or maintain weight; and 3) a BMI below 20. The mean BMI for the eventual sED sample was 22.04, higher than the information provided by Prolific. Although Prolific’s prescreening tools were used to distribute the EAT-26 to participants with BMIs < 20, we calculated BMIs based on self-reported height and weight from the experiment itself (shown in Table 1 and Table S1). This did not affect group inclusion, which was determined using EAT-26 scores. For the HC group, invitations were the sent to people who reported: 1) not having been on a diet before; 2) not restricting food intake to manage weight in the last week; 3) a BMI below 24.9; and 4) not having a diagnosed mental health condition with an ongoing or daily impact. Participants from both groups also needed to be 18–40 years old, fluent in English and have normal or corrected-to-normal vision. 518 individuals completed the initial screening session.

**Table 1:** Group characteristics, including demographics and factor scores.


N	sED		HC				
	
	FEMALE	MALE	FEMALE	MALE			

	40	4	45	11			
	
	M	SD	M	SD	t	df	p

Age	25.30	5.52	26.82	6.09	–1.31	95.94	0.193

BMI	22.04	7.95	22.74	5.88	–0.49	76.79	0.624

Education	15.70	2.79	16.73	3.07	–1.74	96.14	0.085

F1: Eating attitudes	1.05	0.61	–0.83	0.46	17.06	78.01	<.001

F2: Uncertainty sensitivity	0.56	0.94	–0.44	1.03	5.052	95.74	<.001

F3: Anxiety & depression	0.43	1.07	–0.33	0.93	3.72	85.59	0.004


BMI = Body Mass Index. Education refers to years of formal education.

In the second step, people with EAT-26 scores ≥ 20 or less than 10 were invited to complete the full questionnaire battery, listed in the materials section. Questionnaires were presented in a random order. 120 individuals completed the full questionnaire set, with an average completion time of 17.5 minutes. At this stage, we excluded participants who reported taking medication for low mood, anxiety or depression (N = 6). We also remeasured EAT-26 scores. Individuals from the HC group with EAT-26 scores above 10, and individuals from the sED group with EAT-26 scores below 20, were excluded (N = 14).

In the final step, participants who completed the questionnaires and met all inclusion criteria (N = 100) were invited to a decision-making task. To test our experimental predictions, we used a custom patch-leaving task, based on Optimal Foraging Theory (Stephens & Krebs, 1986). The general structure of a patch-leaving task is that participants are presented with an option (a “patch”) that can be exploited for reward. With each round of exploitation, the reward gained from the patch decreases. In light of these declining returns, participants must decide how long to exploit the current patch before switching to a new one that might be better. The particular patch-leaving design used in this experiment combined elements from Constantino and Daw (2015) and Hall-McMaster et al. (2021). Participants controlled a pirate ship sailing to different islands in search of buried treasure. Each block started with a time delay as participants sailed to the first island. After arriving, participants needed to press the spacebar to dig for treasure. This revealed the number of gold medallions added to their treasure chest. Feedback was shown for 1.5s and then disappeared from the screen. Once the feedback disappeared, participants could choose to dig again (spacebar) or set sail from the island (‘s’ key). No time limit was imposed for responses during the task. If the participant chose to dig again, more medallions were added to their treasure chest but it was less than the previous dig. Participants were repeatedly presented with a choice between digging or leaving the current island, until a leave decision was recorded. Once the participant chose to leave, there was a time delay while sailing to the next island. This structure continued for 7.5 minutes at which point the block ended. Throughout the block, the time remaining was shown in the top right corner of the screen.

Reward dynamics in the task were controlled using precise mathematical functions. When first arriving at each island, the reward received for the first dig was drawn from a Gaussian distribution with a mean of 100 and a standard deviation of 10. For each subsequent dig on the island, the reward received was the reward from the previous trial multiplied by a decay constant, *k*:


\[
{\rm Rewar}{{{\rm d}}_{t}} = {\rm Rewar}{{{\rm d}}_{t-{\rm 1}}} * {{k}_{t}}\]

The decay constant used on each trial, *t*, was drawn from a Gaussian distribution with a block specific mean and a standard deviation of 0.07. If the value drawn on a trial was above 1, *k* was set to 1. Participants completed four blocks in total. Across blocks, we manipulated two factors known to influence optimal leaving times: decay rate and travel time. Slow reward decay within patches and short travel time between them reduce the optimal patch residence time (see Constantino & Daw, 2015). In the present experiment, the mean decay constant used in each block was either 0.81 (fast decay) or 0.91 (slow decay). The travel time in each block was either 9s (long travel time) or 2.5s (short travel time). These values were selected on the basis of simulations (Figure S3). Each simulation computed the total reward per block for leaving each patch after a fixed number of actions. Simulations assumed a mean reaction time of 1 second. This allowed us to estimate optimal patch residence times (number of actions per patch) for different decay rate and travel time combinations. We then selected combinations for the experiment that were expected to produce a smooth increase in optimal residence times. One decay/travel time combination was used per block. The block order was randomised for each participant and participants did not have direct experience with the islands or the reward dynamics before starting the first block.

### Statistical Analyses

Data were analysed using appropriate generalised linear models (GLMs), as implemented in *glmmTMB* (Brooks et al., 2017) and *lme4* (Bates et al., 2015) packages. Data-appropriate distributions were selected for each analysis: reaction times and leaving times were analysed using Gamma response distributions, while patch actions (discrete positive numbers) were analysed using Poisson regression. All GLMs used a log link function. Reward data were analysed using a GLM with Gaussian likelihood and identity link function. All models included a random intercept for participant and random slopes for within-subject factors (decay condition, travel time condition). This was determined by estimated models for all combinations of random slopes and selecting the one with lowest Akaike Information Criterion (AIC) and Bayesian Information Criterion (BIC) scores (the two metrics were in agreement in all cases). Statistical significance for each GLM was assessed using the type II Wald *X*^2^ test (using the *car* package; Fox and Weisberg, 2019). Post-hoc analyses including marginal means and slopes estimation were performed using the *modelbased* (Makowski et al., 2020) and *emmeans* (Lenth, 2020) packages. Holm correction was applied to all post-hoc tests to control for multiple comparisons (Holm, 1979). For analyses focusing on RT acceleration, the slope of the change in RT over time was estimated using appropriate GLMs. For example, the following Gamma GLM with a log link function was used to extract remaining-time slopes for each block:


\[
Eq.: RT \sim decay\_fac * travel\_time * group + \left(1 + decay\_fac + travel\_time + \boldsymbol{remaining\_time} | block/id\right)
\]

This approach removes any fixed effects from the data as well as within-subject variability associated with decay and travel time conditions, isolating the remaining time variable for each block.

### Computational modelling

Decisions about whether to exploit or leave a patch were modelled using an approach from Constantino & Daw (2015). Specifically, the model computed the probability of exploiting the current patch at decision *t* using a logistic decision rule:


\[
P{{(exploit)}_{t}} = \frac{1}{1 + exp\left(-\left[c + \beta \left({{r}_{t-1}} - {{T}_{t}}\right)\right]\right)}\]

The core aspect of the expression above is the term *r_t_*_–1_ – *T_t_* which compares *r_t_*_–1_, the last reward received from the current patch, to the current leaving threshold of the model, *T_t_*. The parameter *c* is an intercept that estimates how much people tend to over or under exploit the current patch relative to the reward rate. *β* is a parameter that controls the slope of the logistic function, reflecting participants’ sensitivity to the difference between the leaving threshold and the expected reward for exploiting. The leaving threshold, *T*, was set based on the estimated reward rate for the environment. This estimate was akin to a weighted reward average across all patch decisions in a block up to that point. It was updated following each exploit decision using a simple delta learning rule with a learning rate (*α*):


\[
{{\hat{r}}_{E, t}} = {{\hat{r}}_{E, t-1}} + \alpha \left({{r}_{t}} - {{\hat{r}}_{E, t-1}}\right)\]

Given participants received no immediate reward for decisions to leave a patch, \[
{{\hat{r}}_{E, t}}\] was updated with rewards of 0 following each leave decision. Since reward and no reward might influence participants’ estimate of the reward rate to different extents, we allowed the model to have separate learning rates for rewarded and unrewarded updates (*α* and *α*_1_).

The estimated reward rate for the environment, \[
{{\hat{r}}_{E, t}}\] , was used directly as the leaving threshold:


\[
{{T}_{t}} = {{\hat{r}}_{E, t}}\]

The model had 4 free parameters in total, *α, α_l_, β* and *c*. The parameters were fit for each participant using a scatter-based optimisation solver in MATLAB (version R2021a). The constant, *c*, was constrained between –50 and +50, the parameter *β* was constrained between 0 and +2 and learning rates were constrained between 0 and +1. The estimated reward rate was initialised at 50 at the start of each block. For each trial, the exploit probability was calculated using the logistic decision equation above. One exception to this was on the first decision when arriving at a patch, where participants were forced to exploit and the exploit probability was set to 1–(1e–5), without using the logistic choice equation. On all trials, exploit probabilities were constrained to a maximum value of 1–(1e–5) and a minimum value of 1e–5. The leave probability was calculated as 1–p(exploit). The probability of the choice made on the current trial was stored. The negative log of the choice probabilities were summed to get the negative log likelihood of the model given the data. Model parameters were selected on the basis of minimising the negative log likelihood. Reward rate estimates and latent choice parameters were compared between sED and HC groups using two-tailed independent t-tests.

### Factor analysis

The collected questionnaires (EAT-26, EDE-Q, AAI, CIA, IUS, OCI-R, BDI, STAI-T) were subjected to exploratory factor analysis using oblique rotation (*psych::fa()*; Revelle, 2023). The number of factors was identified at three using the Cattel’s criterion (Cattell, Nelson & Gorsuch, 1967; Cattell-Nelson-Gorsuch test) at three.

### Data and code availability

Raw as well as preprocessed data are available in the associated github repository: https://github.com/ozika/ed-foraging-mcmaster-and-zika.

## Results

To investigate whether individuals with restrictive eating symptoms persist with actions for longer before stopping, we ran a multi-stage online experiment (Figure 1A). Each stage was conducted as a separate online session. In the first stage, prospective participants completed the Eating Attitudes Test (EAT-26; Garner et al., 1982), where higher scores indicate greater concerns about eating. Individuals with scores 20 or above were included in a subclinical ‘eating disorder’ (sED) group and individuals with scores below 10 were included in a ‘healthy control’ (HC) group. People in the sED group did not necessarily have a diagnosed eating disorder and we use this label purely to distinguish the participant group with higher concerns about eating.

In the second stage of the experiment, participants completed a more extensive questionnaire set that again measured eating attitudes, but additionally measured body image, anxiety and depression, sensitivity towards uncertainty and obsessive-compulsive tendencies. The questionnaires used are listed in the methods section under materials.

In the third stage of the experiment, participants completed a decision task (Figure 1B). The task was themed as a treasure collection game, in which participants controlled a pirate ship and sailed to different islands to search for buried treasure. On each island, participants could select between two actions: digging for treasure (an exploit decision) and leaving the island (a leave decision). After an exploit decision, reward feedback appeared showing the number of gold medallions earned. With each successive exploit action, the reward decreased, forcing participants to decide when to leave the current island. When a leave decision was finally made, participants experienced a travel delay while sailing to the next island. This structure continued for 7.5 minutes, at which point the block ended. Participants were shown the remaining block time in the top right corner of the screen. Islands are called patches hereafter for consistency with Optimal Foraging Theory.

Participants completed four blocks in total. Across blocks, we manipulated how quickly rewards declined with each successive exploit decision in a patch (the decay rate), and the travel time between patches. These manipulations took place in a 2 × 2 factorial design, in which the factors were decay rate (fast × slow) and travel time (short × long). Optimal Foraging Theory predicts that faster reward decay and shorter travel times should result in earlier leaving times. We used these predictions to validate the experimental task. Our primary interest was testing the predictions that people with restrictive eating symptoms would spend longer in each patch and exploit patches down to lower levels of reward before leaving.

### Experimental manipulations produced changes in leaving time

We first validated our experimental manipulations. Leaving times are expected to change based on how fast patch values decline and the travel time between patches. Patches that decline in value faster lead to earlier leaving times, as do shorter travel times between patches. Consistent with these expectations, we observed significant main effects of decay rate and travel time on the number of seconds participants spent in each patch before leaving (decay: *X*^2^ (1, *N* = 100) = 278.09, *p* < 0.001; travel time: *X*^2^ (1, *N* = 100) = 110.62, *p* < 0.001). Participants spent fewer seconds in each patch when rewards dropped quickly (M = 11s) compared to slowly (M = 17.4s). Participants similarly spent fewer seconds in each patch when travel times were short (M = 12.1s) compared to long (M = 16.4s). These results confirm that participant behaviour was sensitive to the experimental manipulations.

### Group characteristics

Having validated the experimental task, we examined the characteristics of each participant group. The sED and HC groups were comparable in age, BMI, and education. Formal statistics are presented in Table 1. Gender counts were not significantly different between groups (*X*^2^ (1, *N* = 100) = 0.94, *p* = 0.331). Mean scores on all questionnaires administered differed between the groups (see Table S1). This included significant differences in the dieting and oral control subscales of the EAT-26 between groups, which contain items that probe restrictive eating behaviours (Gardner et al., 1982; Berland et al., 1986). As multiple questionnaires were used to measure similar constructs (e.g. concerns about eating, anxiety), we performed a factor analysis to identify transdiagnostic factors spanning across specific measures (Wise et al., 2023). This resulted in three factors: Factor 1 (F1: Eating attitudes) loaded mainly on questionnaires concerned with eating attitudes and appearance anxiety (EAT-26, EDE-Q, AAI). Factor 2 (F2: Uncertainty sensitivity) was characterised by questionnaires measuring intolerance to uncertainty and partially by obsessive-compulsive tendencies (IUS and OCI-R). Factor 3 (F3: Anxiety and depression) loaded predominantly on trait anxiety and depression measures (STAI-T, BDI). Scores on all three factors differed between the groups. Formal statistics are presented in Table 1 and full factor loadings and presented in Figure S1. These results confirm that the experimental procedure was successful in recruiting two distinct participant groups, who differed in their levels of concern about eating and self-reported restrictive eating symptoms.

### Subclinical ED and control groups showed comparable leaving behaviour

**Figure 2:**
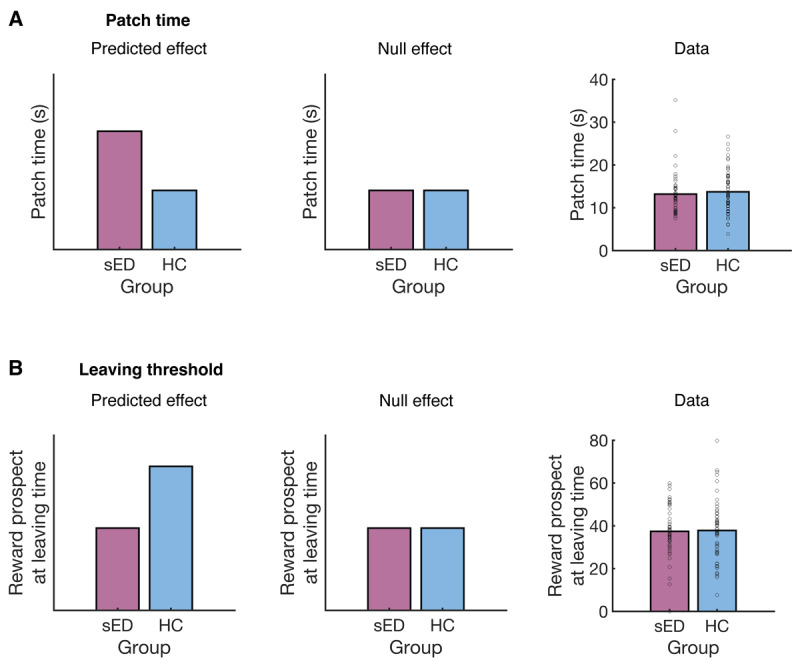
Theoretical predictions and empirical results. **A:** Patch residence time. The left panel shows the predicted group difference, in which the subclinical eating disorder (sED) group is expected to spend longer in each patch before deciding to leave. The middle panel shows the expected data profile in the case of no group difference. The right panel shows the empirical patch residence time for the sED and HC groups. **B:** Leaving threshold. The left panel shows the predicted group difference, in which the reward prospect for an exploit decision is lower at the time of leaving for the sED group. As patch rewards decrease monotonically, the reward prospect for exploiting is a useful measure of current patch value, and thus the reward level/threshold at which patches are abandoned. The middle panel shows the expected data profile in the case of no group difference. The right panel shows the empirical leaving threshold for the sED and HC groups. A-B: Dots show data from individual participants.

We next tested our experimental predictions: 1) that the sED group would spend longer in each patch before leaving and 2) that the sED group would exploit each patch down to a lower reward level before leaving. Contrary to the first prediction, we did not detect a significant main effect of group on the number of seconds spent in each patch (M_sED_ = 13.10s, SD_sED_ = 5.14, M_HC_ = 13.72s, SD_HC_ = 4.94, *X*^2^ (1, *N* = 100) = 0.65, *p* = 0.420, Figure 2A). Contrary to the second prediction, we did not detect a significant main effect of group on the prospective patch reward, at the time participants decided to leave (M_sED_ = 37.75 points, SD_sED_ = 11.87, M_HC_ = 37.82 points, SD_HC_ = 13.96, *X*^2^ (1, *N* = 100) = 0.029, *p* = 0.864, Figure 2B). As patch rewards decline monotonically, the prospective reward for exploiting is a good approximation of a patch’s current reward level. The pattern of results above did not change when considering more specific interactions. The time in each patch was not influenced by an interaction between group and travel time (*X*^2^ (1, *N* = 100) = 1.35, *p* = 0.246), nor by an interaction between group and decay rate (*X*^2^ (1, *N* = 100) = 0.63, *p* = 0.427). The prospective patch reward was not influenced by an interaction between group and travel time (*X*^2^ (1, *N* = 100) = 0.68, *p* = 0.409), nor by an interaction between group and decay rate (*X*^2^ (1, *N* = 100) = 0.017, *p* = 0.895). These results suggest that the sED and control groups showed comparable decisions about when to disengage from each option.

### Subclinical ED and control groups had comparable choice parameters

Even in the absence of overt behavioural differences, it is possible the sED and HC groups differed in the latent processes underlying their decisions (see Pike et al., 2023). To test this possibility, we performed a computational modelling analysis. The analysis modelled decisions to exploit or leave a patch based on the Marginal Value Theorem (Charnov, 1976), which compares the instantaneous patch reward to the average reward rate for the environment. Following a previous established approach (Constantino & Daw, 2015), we assumed that participants formed an internal estimate of the average reward rate, based on feedback during the task. The comparison between average reward rate and the instantaneous patch reward was implemented using a logistic choice function. The model had four free parameters in total, *α, α_l_, β* and *c*. The parameters *α, α_l_* were learning rates used to update the estimated reward rate after an exploit decision (*α*) or a leave decision (*α_l_*). The parameter *β* captured participants’ sensitivity to the difference between the reward rate and instantaneous patch rewards. The parameter *c* was a constant that estimated how much people tended to over or under exploit the current patch relative to the reward rate. More details about the model are presented in the methods. The computational modelling analysis revealed that internal reward rate estimates were not significantly different between groups (M_sED_ = 63.82, SD_sED_ = 10.52; M_HC_ = 64.97, SD_HC_ = 10.19; *t*(98) = –0.55, *p* = 0.583). This suggests that both groups had a similar patch leaving threshold. Moreover, best fitting parameters values did not significantly differ between groups: *α* (M_sED_ = 0.13, SD_sED_ = 0.12; M_HC_ = 0.15, SD_HC_ = 0.12; *t*(98) = –0.70, *p* = 0.486), *α_l_* (M_sED_ = 0.02, SD_sED_ = 0.08; M_HC_ = 0.01, SD_HC_ = 0.05; *t*(98) = 0.68, *p* = 0.50), *β* (M_sED_ = 0.20, SD_sED_ = 0.12; M_HC_ = 0.19, SD_HC_ = 0.09; *t*(98) = 0.36, *p* = 0.721), *c* (M_sED_ = 4.62, SD_sED_ = 6.64; M_HC_ = 4.57, SD_HC_ = 6.00; *t*(98) = 0.04, *p* = 0.971). These results suggest that latent parameters underpinning patch choices were comparable between the sED and control groups.

### The subclinical ED group showed increasing response vigour

**Figure 3:**
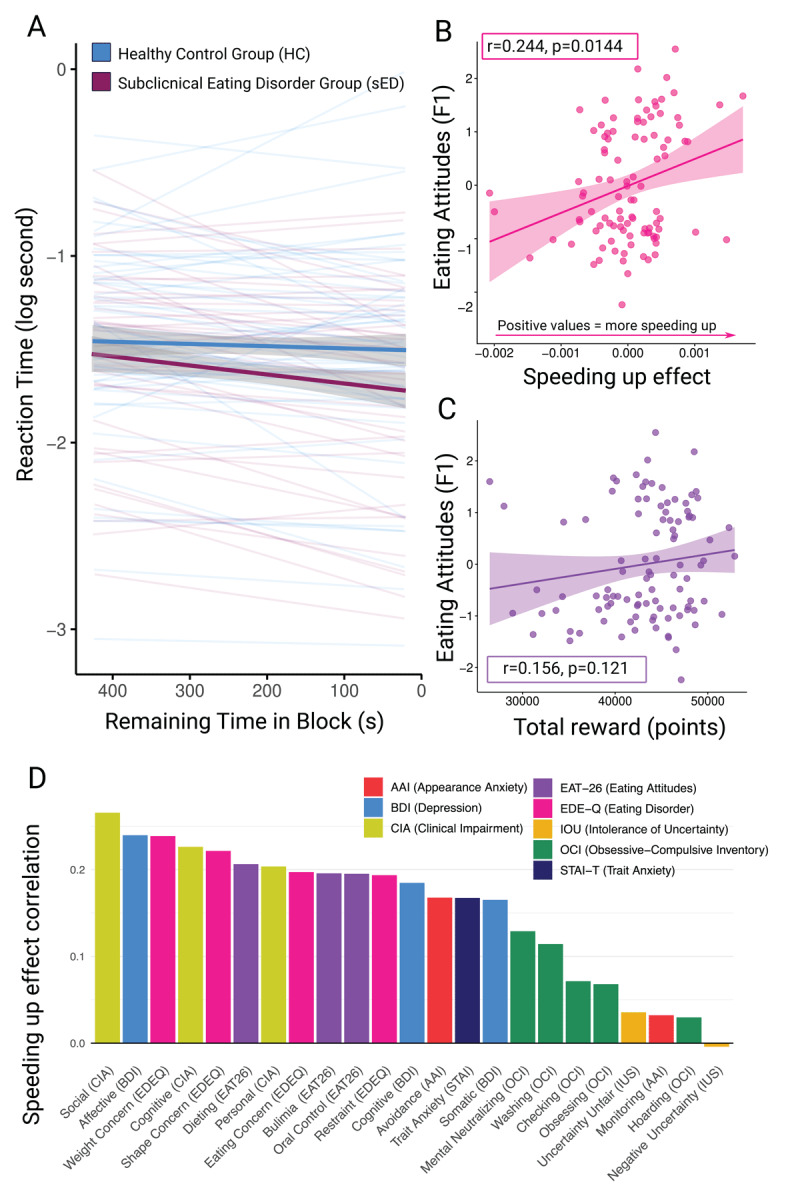
Increasing vigour, eating attitudes and reward. **A.** Increasing vigour effect by group. The sED group (shown in maroon) was found to decrease their reaction times with the approaching end of each block, while there was no trend in the HC group (shown in blue). The thick lines represent posterior marginal means with 95% confidence intervals. The thin lines represent estimates for each individual. **B.** Spearman correlation between the Eating Attitudes factor and the slope of the increasing vigour effect. More positive values represent higher reduction in reaction times towards the end of the block. **C.** Spearman correlation between the Eating Attitudes factor and the amount of reward accumulated throughout the task. All panels in this plot depict individual data for 100 individuals. **D.** Questionnaire subscales and their correlation with increasing motor vigour under time pressure. The y-axis shows the magnitude of the correlation coefficient. The x-axis shows established subscales for each questionnaire. Colours denote subscales from the same questionnaire.

Next, we performed a set of exploratory analyses, to ask whether there were alternative markers distinguishing the sED and HC groups. Based on previous reports of faster decision-making and motor function in AN compared to HCs (King et al., 2016; Pieters et al., 2003), we were motivated to examine potential differences in reaction times (RTs). One strong group difference was evident in how much participants’ RTs changed as a function of remaining time in each block (total block length: 720 sec; visible to participants). We observed a significant interaction between group and remaining time in the block on choice RTs (*X*^2^(1, *N* = 100) = 49.50, *p* < 0.001, Figure 3A). Bootstrapping tests indicated this effect was highly robust. When selecting 60–80% of the participants at random and re-computing the test statistic across 500 permutations, the bootstrapped 95% confidence interval for *X*^2^ was [13.85, 70.97]. This means even under conservative assumptions of a reduced sample size and the minimum expected test statistic, the interaction between group and block time was still highly significant (*X*^2^(1, N = 70) = 13.85, *p* < 0.001, Figure S4). Follow up tests comparing the change in RTs between groups revealed that the sED group showed a stronger reduction in RTs with increasing block time, compared to the HC group (B_HC_ = –1.91e–04, B_sED_ = –5.55e–04, *B_sED-HC_* = –3.64e–04, SEM = 5.03e–05, *z_ratio_* = –7.24, *p* < 0.001). Providing some intuition, the HC group took on average 0.37s to respond at the end of the block compared to 0.42s at the beginning of the block, while the sED group took about 0.27s to respond at the end compared to 0.39s at the beginning (coded as: beginning = 1st–10th remaining time quantile; end = 91st–100th remaining time quantile). The difference in RT between HC and sED groups was already significant in the first 10% of trials (Welch two sample t-test, *t*(79) = –2.51, *p* = 0.014).

To assess whether this effect was specific to eating attitudes, we compared regression models that used either the pre-assigned group membership (HC/sED) or the estimated factors F1 (eating attitudes), F2 (uncertainty sensitivity) or F3 (anxiety and depression) as the individual difference variable. The model that best predicted RTs across time was the model using F1 (eating attitudes), which was evident in a lower AICc score (–25282.2) compared to the other models (group: –25272.6, F2: –25227.8, F3: –25207.0). This indicates that eating attitudes were the best predictor of the decreasing RT effect. This finding was consistent with the *X*^2^ values for the interaction of the factor scores (F1–3) with remaining time within each model. The F1:remaining-time interaction was qualitatively highest (*X*^2^(1, *N* = 100) = 73.38, *p* < 0.001). The F2 interaction with remaining time was also significant but had a qualitatively lower *X*^2^ value (*X*^2^(1, *N* = 100) = 18.18, *p* < 0.001). The interaction of remaining time and F3 was not significant (*X*^2^(1, *N* = 100) = 1.66, *p* = 0.197; Figure S2). Spearman correlation results were consistent with this pattern, indicating a robust association between higher eating concerns (F1 scores) and RT acceleration, *r_s_*(98) = 0.244, *p* = 0.014 (Figure 3B).

Next, we examined whether the speeding up effect was related to a broad range of ED symptoms, or a specific ED subtype. We approached this in two ways. First, we computed individual scores for all 100 participants on predefined subscales within each questionnaire (e.g. dieting, bulimia and oral control in the EAT-26) and correlated them with the speeding up effect. Specifically, we calculated Spearman correlation coefficients between the distribution of scores for each subscale and the corresponding distribution of RT acceleration slopes from the linear mixed effects model. The highest correlation coefficients were related to multiple subscales of the ED questionnaires (EAT-26, EDE-Q, CIA), and the affective subscale of the depression questionnaire BDI (Figure 3D). The ED subscales with high, positive correlation coefficients reflected a broad range of ED symptomatology, including shape and weight concerns, dieting, oral control, bulimia, as well as social, cognitive and personal impairment due to ED behaviours. We then repeated the analysis for each individual item across all questionnaires. Items from a range of ED questionnaires and symptom dimensions were again among the highest correlation coefficients (Figure S5). These results suggest that speeding up under time pressure is associated with a broad range of ED symptoms, related to multiple traditional diagnostic categories (anorexia, bulimia and binge eating disorder).

In a final set of exploratory analyses, we tested whether group membership and continuous restrictive eating symptoms were related to performance. The sED group earned more reward on average than HCs (R_sED_ = 44067, R_HC_ = 42530), although this increase was not statistically significant, *X*^2^(1, N = 100) = 2.03, *p* = 0.15). When looking at continuous variation in restrictive eating symptoms, we did not detect a significant spearman correlation between the F1 factor and overall reward (*r_s_*(98) = 0.156, *p* = 0.121; Figure 3C). Together, the results in this section indicate that a robust difference between the sED and HC groups was how much their responses accelerated as the time in each block decreased. This speeding up under time pressure was not specific to restrictive ED symptoms. Instead, it was positively related to a broad range of ED characteristics and symptoms.

## Discussion

The present experiment aimed to test the hypothesis that individuals with subclinical restrictive eating disorder (sED) tendencies take longer to disengage from actions or activities that are no longer rewarding. To measure time until disengagement, we used a theoretical framework called Optimal Foraging Theory (Stephens & Krebs, 1986), in which a single option is exploited at a time as its reward decreases, and people must decide when to disengage from it. We predicted that a subclinical ED group would (1) spend longer exploiting each option and (2) that each option’s reward prospect would be lower when deciding to leave, in comparison to healthy controls (HCs). Contrary to these predictions, the sED group showed comparable exploitation times and reward prospects at the point of leaving. Latent choice variables and parameters were also comparable between groups. Unexpectedly, exploratory analyses identified robust group differences in the RT domain. RTs for the sED group decreased throughout each block, significantly more than the HC group. Follow-up analyses showed that eating attitudes was the strongest predictor of this effect. Moreover, eating attitudes reflected a broad range of ED symptoms associated with all three traditional diagnostic categories (anorexia, bulimia and binge eating disorder). Increases in motor vigour were positively related to multiple ED subscales, including shape and weight concerns, dieting, oral control and bulimia, as well as self-reported impairment in social, cognitive and personal domains due to ED features. Together, these factors indicate that ED symptoms are associated with increasing response vigour under time pressure and that this could be a transdiagnostic marker of eating disorders, which includes symptoms from multiple ED subcategories.

Increasing motor vigour in the sED group is consistent with past research reporting faster RTs in acute AN, which could indicate more efficient processing (King et al., 2016) or reduced dependence on reward that lowers deliberation time and results in more automatic responding (Steinglass & Walsh, 2016). The sED group might also have been calibrating motor vigour to maximise reward rate (Sukumar et al., 2024; Yoon et al., 2018). This interpretation can be explained with reference to Yoon et al. (2018), who demonstrated a normative relationship between motor vigour and reward rate during foraging. In their framework, the average reward rate depends on both patch leaving times and the vigour with which one moves to the next patch. Hence, motor vigour itself is an explicit variable available for optimisation. Travel times between patches were fixed in the present experiment. However, high-level principles from Yoon et al. (2018) can be applied: participants could increase their reward rate by increasing their motor vigour within patches. Indeed, further analysis showed that participants who sped up more within each patch tended to earn higher reward overall, *r_s_*(98) = 0.243, *p* = 0.015. Future research could consider utilising additional computational frameworks to understand the mechanisms that connect increasing motor vigour and sED symptomatology, such as foraging-adapted drift diffusion models (Davidson & El Hady, 2019).

One intriguing aspect of accelerating motor vigour was its transdiagnostic nature. Speeding up under time pressure was related to a broad set of eating attitudes (the F1 factor), ED subscales, and self-reported impairments due to ED tendencies. Based on this, it is interesting to consider whether there could be a common denominator underpinning this effect. Anxiety disorders commonly co-occur across multiple ED diagnoses (Swinbourne & Touyz, 2007) and their subclinical counterparts (Touchette et al., 2011). One possibility could be that individuals with greater eating concerns were more prone to becoming stressed or anxious about the reducing time in each block. One argument against this interpretation is that weighted anxiety and depression scores (the F3 factor) were not significant predictors of the difference in RT between the groups (Figure S2D). Previous research has shown that intolerance for uncertainty and obsessive-compulsive tendencies also tend to be elevated in multiple ED diagnoses compared to HCs (Brown et al., 2017; Drakes et al., 2021; Kesby et al., 2017). However, correlation coefficients between the speeding up effect and intolerance for uncertainty, as well as obsessive-compulsive tendencies, were the among the lowest measured. The data from our experiment suggest that speeding up under time pressure was related to a wide range of ED symptoms, but did not extend into several domains that can co-occur with EDs, such as intolerance for uncertainty or obsessive-compulsive tendencies.

From a broader perspective, a recurring theme in previous studies on restrictive EDs has been that cognition and behaviour is rigid and inflexible to change (Filoteo et al., 2014; Steinglass et al., 2006; Wu et al., 2014; Rizk et al., 2015). Computational studies have proposed several possible mechanisms for this maladaptive persistence, including reduced goal-directed control (Foerde et al., 2021; Onysk & Seriès, 2022), reduced risk aversion in disorder-relevant contexts (Jenkinson et al., 2023), as well as reduced exploratory behaviour and increased perseveration (Filoteo et al., 2014). A recent study by Pike et al. (2023) suggests the picture is more nuanced, demonstrating more flexible adjustments in learning rate between task environments in women who had recovered from AN. The present results provide a second example of an ED-related group exhibiting more flexible adjustments in behaviour than controls. These recent examples highlight potential domains of increased flexibility for future research on restrictive EDs.

We predicted that individuals with restrictive ED symptoms would show increased persistence and reduced decision thresholds in a neutral patch-leaving task, compared to HCs. However, we did not see evidence for these predictions. One possible reason could be the abstract decision task used, which was not specific to EDs. This is relevant because past research indicates that, although some cognitive changes in restrictive EDs can be detected in abstract settings (e.g. Foerde et al., 2021), other changes are visible in disorder-relevant settings (Jenkinson et al., 2023; Onysk & Seriès, 2022). In addition, there is good reason to be critical about the hypothesis that individuals with restrictive eating disorders are more persistent in general, without considering how persistence is regulated in different contexts. When eating, for example, individuals with AN clearly do not persist in their consumption, with reduced calorie intake and a slower eating rate (Sysko et al., 2005), although mealtimes themselves can be longer due to more non-ingestive behaviours, such as moving food around (Tappe et al., 1998). The implication is that our data do not preclude changes in an internal threshold for behavioural adaptation in restrictive EDs, in contexts where decisions are relevant to ED symptoms (such as choice contexts involving food, exercise or body image). Future research could therefore examine the predictions from this experiment in a task adapted to EDs, with the predicted direction of changes in decision threshold set on the basis of relevant symptoms. Another possible reason for not detecting differences in persistence or decision thresholds in this experiment could be the sample tested. The predictions for this experiment were primarily motivated on the basis of studies indicating maladaptive persistence in individuals with acute AN or individuals who had recovered from AN (Filoteo et al., 2014; Steinglass et al., 2006; Wu et al., 2014; Rizk et al., 2015). The intensity and variability of symptoms in our subclinical sample was plausibly different from these formal clinical groups. It is therefore possible that the predicted effects might still be observed in restrictive ED patients with a formal diagnosis.

To conclude, the present experiment did not find evidence for a change in the decision threshold used to adapt behaviour in restrictive EDs. These findings do not rule out a change in decision threshold in contexts relevant to ED symptoms, such as food, exercise and body image. The findings neither rule out such a change in individuals with a formal restrictive ED diagnosis. Overall, this experiment showed that subclinical individuals with heightened ED concerns showed a robust increase in motor vigour over time that was higher than HCs. Eating attitudes were the strongest predictor of speeding up under time pressure, more so than uncertainty sensitivity or anxiety and depression symptoms (see Figure S2). Moreover, this effect was related to a wide range of ED symptoms from multiple traditional diagnostic categories (see Figure 3, S5). We emphasise that these findings were unexpected, exploratory results and should be replicated for confirmation. Together, the findings suggest that increasing response vigour under time pressure may be a transdiagnostic marker of ED tendencies.

## Additional File

The additional file for this article can be found as follows:

10.5334/cpsy.130.s1 Supplementary Information. Figures S1 to S5 and Table S1.
