# Supplementary material for: Increasing Response Vigour Under Time Pressure as a Transdiagnostic Marker of Eating Disorders
Source: Comput Psychiatr. 2025 Aug 14;9(1):124–41. doi: 10.5334/cpsy.130 (PMC12352398; doi:10.5334/cpsy.130)
Supplement: Supplementary Information. — Figures S1 to S5 and Table S1. [file cpsy-9-1-130-s1.pdf]

Supplementary Information

"Increasing response vigour under time pressure as a transdiagnostic marker of eating disorders"

Sam Hall-McMaster* [1,2] and Ondrej Zika* [1,3,4,5]

* Both authors contributed equally

Factor analysis

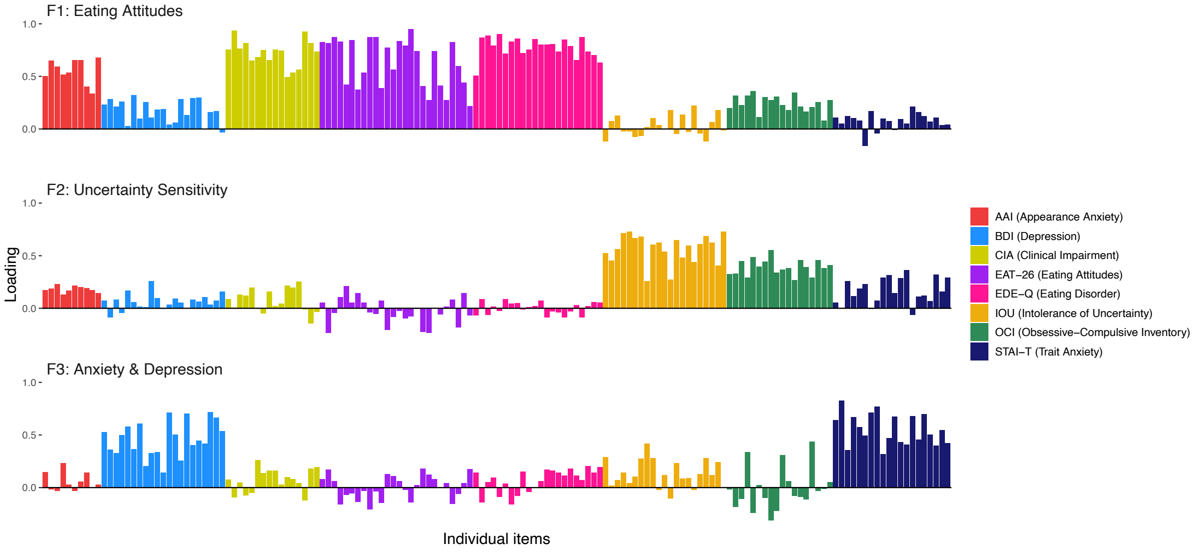

Figure S1. Exploratory factor analysis identified three factors: F1 (Eating Attitudes), F2 (Uncertainty Sensitivity), and F3 (Anxiety & Depression). The y-axis represents loadings of each item on a respective factor dimension, with every bar representing a unique questionnaire item. Colours represent items from the same questionnaire. The questionnaires included the Appearance Anxiety Inventory (AAI; Veale et al., 2014), Beck Depression Inventory (BDI-II; Beck et al., 1996), Clinical Impairment Assessment (CIA; Bohn & Fairburn, 2008), Eating Attitudes Test (EAT-26; Garner et al., 1982), Eating Disorder Examination Questionnaire (EDE-Q; Fairburn & Beglin, 2008), Intolerance for Uncertainty Scale (IUS; full version; Buhr & Dugas, 2002), Obsessive-Compulsive Inventory (OCI; Foa et al., 2002), and the State-Trait Anxiety Inventory (STAI; Spielberger, 1983).

Extended group characteristics

Table S1: Extended group characteristics, including demographics and factor scores

	sED		HC				
	Female	Male	Female	Male			
N	40	4	45	11			
	M	SD	M	SD	t	df	p
Age	25.3	5.52	26.82	6.09	-1.31	95.94	0.193
BMI	22.04	7.95	22.74	5.88	-0.49	76.79	0.6243
AAI	23.5	7.74	8.29	6.43	10.47	83.12	<0.001
EAT-26	36.05	10.73	3.12	2.52	19.93	46.73	<0.001
EAT-26 (Bulimia)	6.91	3.54	0.05	0.23	12.84	43.28	<0.001
EAT-26 (Dieting)	22.61	6.62	1.7	1.57	20.52	46.82	<0.001
EAT-26 (Oral control)	6.52	4.65	1.38	1.46	7.08	49.69	<0.001
EDE-Q	89.89	17.6	15.75	14.23	22.71	81.67	<0.001
CIA	25.61	9.2	3.34	4.1	14.934	56.35	<0.001
IUS	63	16.69	47.39	14.98	4.86	87.31	<0.001
OCI	24.36	12.83	10.27	9.16	6.16	74.97	<0.001
BDI	19.18	8.84	7.09	7.19	7.36	82.04	<0.001
STAI-T	55.98	9.54	41.68	11.51	6.79	97.7	<0.001
**Factors**							
F1: Eating attitudes	1.05	0.61	-0.83	0.46	17.06	78.01	<0.001
F2: Uncertainty sensitivity	0.56	0.94	-0.44	1.03	5.05	95.74	<0.001
F3: Anxiety & depression	0.43	1.07	-0.33	0.93	3.72	85.59	<0.001

Abbreviations: BMI = Body Mass Index, AAI = Appearance Anxiety Inventory (Veale et al., 2014), EAT-26 = Eating Attitudes Test (Garner et al., 1982), EDE-Q = Eating Disorder Examination Questionnaire (Fairburn & Beglin, 2008), CIA = Clinical Impairment Assessment (Bohn & Fairburn, 2008), IUS = Intolerance of Uncertainty Scale (Buhr & Dugas, 2002), OCI = Obsessive Compulsive Inventory (Foa et al., 2002), BDI = Beck Depression Inventory (Beck et al., 1996), STAI-T = State Trait Anxiety Inventory - Trait Anxiety (Spielberger, 1983).

Factor scores and speeding up effect

In the main text we report an interaction effect, in which the sED group exhibited a significantly faster RT acceleration within each block. Model comparison and X^2 statistics for the factor*remaining-time interaction suggested that eating attitudes had the strongest impact on the acceleration effect. To further assess this, we performed a median split for each factor and re-plotted the data (Fig. S2). The figure shows RT plots split by group (as in the main text) and split by the median for each factor. Visual inspection of the figure confirms the statistical result: eating attitudes appear to be associated with RT acceleration most prominently.

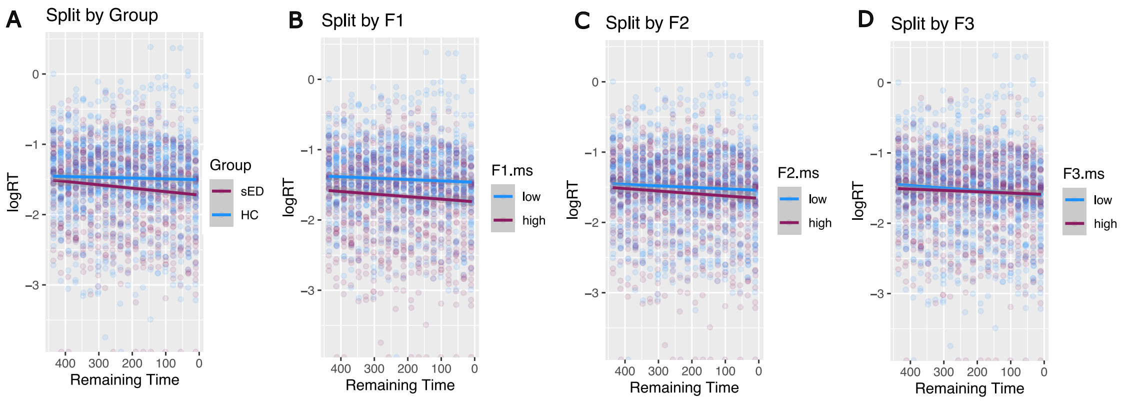

Figure S2. Increasing vigour effect as a function of group assignment (A), or median split by the factors Eating attitudes (B), Uncertainty sensitivity (C) and Anxiety & Depression (D). In each panel, the more severe group (i.e. individuals with factor scores above median value) is shown in maroon while the less severe group is shown in blue. The left-most panel corresponds directly to Figure 3A. All demonstrate the relationship between reaction times (y-axis = log seconds) over time (x-axis = remaining time in a block). The thick lines represent posterior marginal means with 95% confidence intervals. The dots represent individual participant data for each time bin.

Simulation results

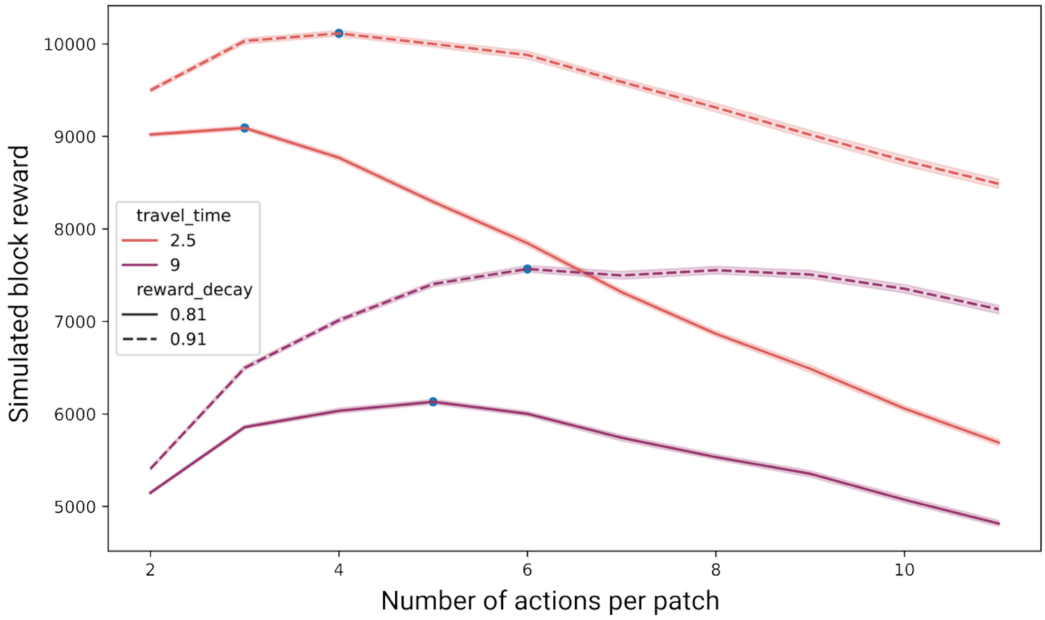

Figure S3. Simulated block reward for taking a fixed number of exploit actions per patch in each condition. Simulations are shown for different travel times (2.5s = orange; 9s = purple) and reward decay rates (0.81 = solid lines; 0.91 = dashed lines). A dot at the top of each simulated curve represents the optimum, assuming response times of 1s.

Bootstrapping analysis of the main exploratory effect

Bootstrapping analysis was performed to assess the robustness of the main exploratory result (i.e. the interaction between group and remaining block time on reaction time). Across 500 iterations, we randomly selected a subsample comprising 60-80% of the participants and re-ran the main statistical model, recording the X^2 value for the interaction. This resulted in a 95% bootstrapped 95% confidence interval for X^2 of [13.85, 70.97]. This indicates the effect was robust. Even if we assume a reduced sample size and the minimum expected test statistic, the interaction remained significant ($X^2(1, N=70)=13.85$, p<0.001).

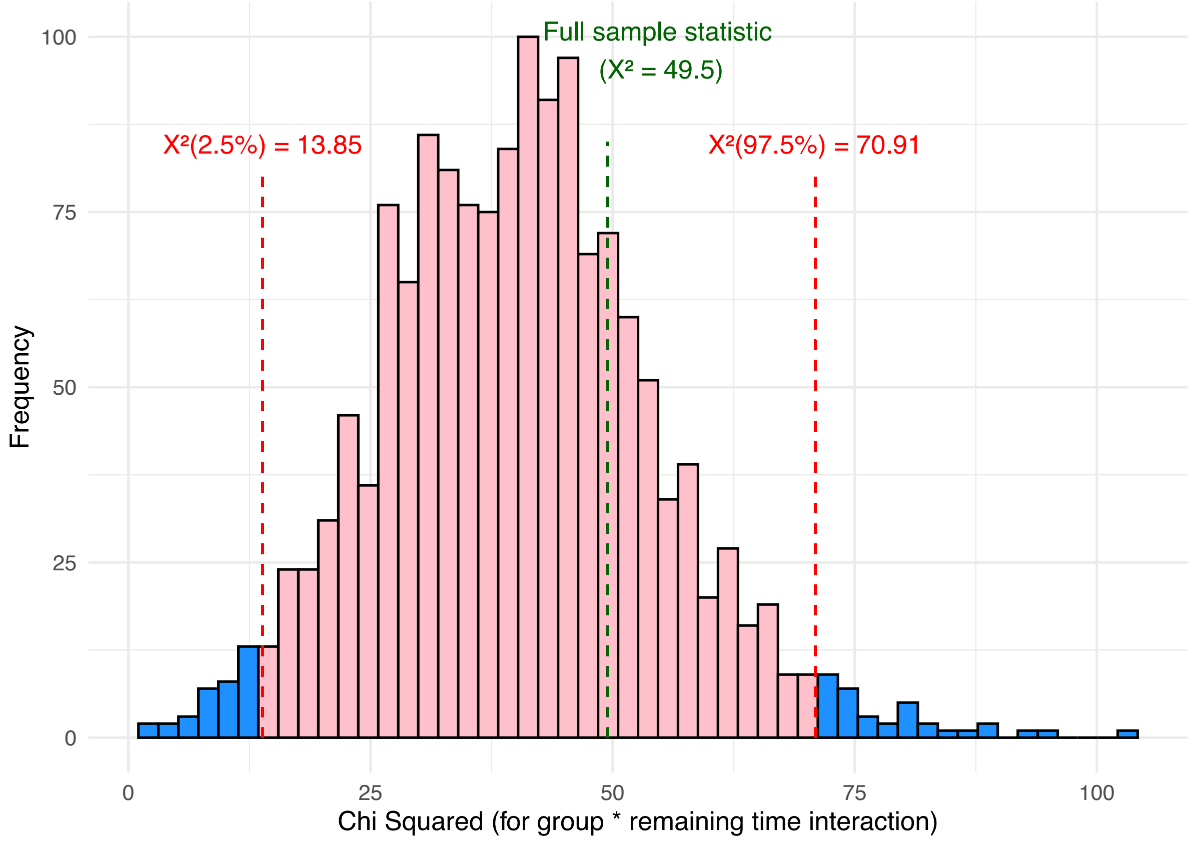

Figure S4. Distribution of bootstrapped Chi Squared values for the interaction term group*remaining_time

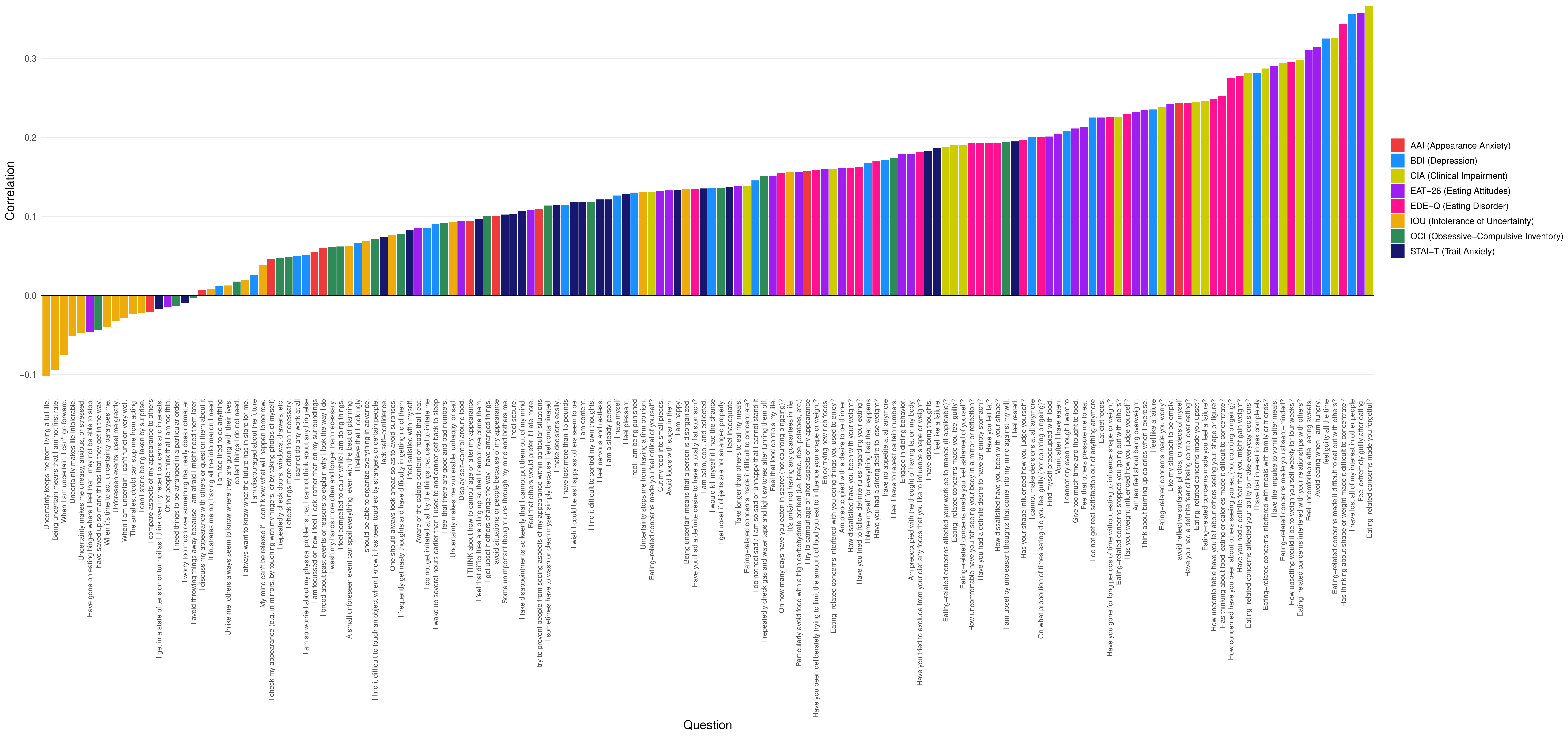

Figure S5. Spearman correlations between the speeding up effect and all questionnaire items. Colours denote items from the same questionnaire. The questionnaires included the Appearance Anxiety Inventory (AAI; Veale et al., 2014), Beck Depression Inventory (BDI-II; Beck et al., 1996), Clinical Impairment Assessment (CIA; Bohn & Fairburn, 2008), Eating Attitudes Test (EAT-26; Garner et al., 1982), Eating Disorder Examination Questionnaire (EDE-Q; Fairburn & Beglin, 2008), Intolerance for Uncertainty Scale (IUS; full version; Buhr & Dugas, 2002), Obsessive-Compulsive Inventory (OCI; Foa et al., 2002), and the State-Trait Anxiety Inventory (STAI; Spielberger, 1983).
